# Monitoring of the Static and Dynamic Displacements of Railway Bridges with the Use of Inertial Sensors

**DOI:** 10.3390/s20102767

**Published:** 2020-05-12

**Authors:** Piotr Olaszek, Ireneusz Wyczałek, Damian Sala, Marek Kokot, Andrzej Świercz

**Affiliations:** 1 Road and Bridge Research Institute, 03-302 Warsaw, Poland; 2 Institute of Civil Engineering, Poznan University of Technology, 60-965 Poznań, Poland; ireneusz.wyczalek@put.poznan.pl; 3 ADAPTRONICA Sp. z o.o., 05-092 Łomianki, Poland; damian.sala@adaptronica.pl (D.S.); marek.kokot@adaptronica.pl (M.K.); 4 Institute of Fundamental Technological Research, Polish Academy of Sciences, 02-106 Warsaw, Poland; aswiercz@ippt.pan.pl

**Keywords:** bridge monitoring, dynamic and static displacements, inertial sensors, total station, indirect measurement

## Abstract

In the case of the monitoring of bridges, the determination of vertical displacements is one of the most important issues. A new measuring system has been developed and implemented for assessment of railway bridges based on measurements of the structural response to passing trains. The system uses inertial sensors: Inclinometers and accelerometers that do not need any referential points. The system records signals related to the passage of a train over a monitored bridge. The signals from inclinometers before the train’s entry are used to determine the static movement. Integrated signals from inclinometers and accelerometers are used to determine dynamic displacements when the train goes through the bridge. Signals from inclinometers are used to determine the so-called “quasi-static” component of the displacement and signal from the accelerometer to determine the dynamic component. Field tests have been carried out on a viaduct along a high-speed railway line. Periodic comparative measurements are carried out using a Total Station to verify static measurements and using inductive sensors to verify dynamic measurements. Tests of the system carried out so far have proven its usefulness for monitoring bridges in a high-speed railway (up to 200 km/h) with high accuracy while determining dynamic displacements.

## 1. Introduction

For assessment of railway bridges, particularly those located at high-speed train lines, theoretical analyses, based on experimental research, are of major importance [1,2,3]. Bridge statics has been analyzed on the basis of geodetic measurement techniques for a long time. For detecting static vertical displacements, geometric leveling was mainly applied, and for horizontal ones—angular techniques. Thanks to the development of technology, geodetic methods have been enriched with very precise distance measurements, and automation of measurements and calculations [4,5]. A significant contribution to the development of monitoring was Global Navigation Satellite Systems (GNSS)-based surveying techniques, mainly in the Real Time Kinematic (RTK) [6] and Precise Point Positioning (PPP) methods [7]. Despite their lower accuracy when compared to traditional methods, GNSS surveys have the advantage that they can be used independently of humans in a quasi-continuous manner, with a real frequency of up to 10 Hz. Interferometric Synthetic Aperture Radar (InSAR) has also become a useful tool for deformation monitoring of large bridges, but its accuracy depends on the construction orientation and number of scenes used, which may be a limiting factor [8,9]. Thanks to the increasing possibilities of digital photography, it is used in situations in which quick, simultaneous measurement must cover a greater number of control points located in different places of the tested object [10,11,12].

Dynamic tests and continuous monitoring systems of bridges are used to [13,14,15,16]:

- validate design conceptions,
- evaluate condition state,
- assess behavior under high-speed train loadings,
- carry out damage detection based on vibration analysis.

In practically all these cases, acceleration measurements are carried out. For the first two and for a part of the third case, it may be useful or even necessary to measure the vertical displacement of bridge girders. While the measurements of the accelerations are relatively simple to carry out, the measurements of displacements in an automatic, unattended mode create considerable difficulties. Major difficulties are encountered especially in the case of structures with restricted or impossible access to the area under them (bridges over busy roads, railway lines, or rivers). In such cases, it is virtually impossible to use inductive gauges. Then, the best solution is using remote systems such as a ground-based microwave interferometer (GBMI) or vision-based vibration measurement systems [17,18,19]. The alternative is using inertial sensors, which do not need any referential points. The Polish scientific and technological consortium led by the Road and Bridge Research Institute has attempted to develop its own technology, first presented in [20,21] at an early stage of development. The first objects of its applications are steel railway bridges.

Many researchers carry out work related to the use of inertial sensors to indirect measurement of dynamic displacements. They present works on using accelerometers to measure both accelerations and displacements of bridges under dynamic loads. A disadvantage of this method is the necessity of double integration of an acceleration signal, which can lead to considerable errors in estimating the displacements. There are various methods of correcting such errors suggested in [22,23,24,25,26], but it is generally not possible to accurately estimate the displacements on the basis of accelerations without additional measurements, such as strain gauge measurements [27,28]. In [29], another solution was presented in which accelerometers were used to define the angles of rotation in place of inclinometers, and the deflection was then determined. In [30], the research studies of the dynamic displacement measurement system using one passive-servo electromagnetic-induction velocity sensor are presented. The results of the system tests at the shake table test show that direct reference-free displacement errors are less than 10% when compared to linear-variable-differential-transducer (LVDT) measurements.

A number of articles related to the use of inclinometers for monitoring automatic displacements of bridge structures were published. Most of those papers are limited to measurements under static load, but not all articles contain information on the accuracy achieved. The paper [31] presents a static test of a post-tensioned concrete highway bridge (main span is 150 m), and the deflection was about 40 mm with an error of less than 5%. In reference [32,33], the method was applied on two bridge spans (length of 24 and 30 m), and the maximum deviation for a deflection of about 16.0 mm reached 1.2–1.6 mm. The article [34] presents tests with the use of high-precision inclination sensors and results concerning two structures of extreme spans (160 versus 18 m) and maximum measured deflection values (175 versus 4 mm). The deviation between the inclinometer system and the reference measurements was 1.25 versus 0.12 mm (0.7% versus 3.0%). Hou [35] presents the use of inclinometers to determine deflections at a bridge with a 64 m span length during static and dynamic load tests. During the static test, deflections were about 22 mm and relative errors were within 2%.

Hem [36] presents the use of inclinometers for tests of a railway steel arch bridge (100 m span length); the train’s maximum speed equaled 120 km/h, with deflections of about 3.5–3.8 mm, and the deviations to the reference methods ranged from 2.9% to 4.5%.

Works using inclinometers to determine displacements under dynamic load and high-speed train passages are presented in [37]. The inclinometers were installed at a 30 m span length, the speed of the train was about 200 km/h, and the deviations to the reference methods ranged from 3.0% to 8.3%. In addition, Martí-Vargas [38] presents discussion comments to the method presented.

The new method presented here is based on the integration of the signals from inclinometers and accelerometers. Solutions using measurements of inclination and acceleration are also presented in the literature. In [39], the main sensor is the accelerometer that is used to evaluate both inclination and three-dimensional acceleration, both dynamic and static. The system may be successfully adopted to detect rockfall events on protection barriers, as well as to monitor landslides or the integrity of structures like bridges and buildings. A way of integrating the signals from an inclinometer and an accelerometer in order to determine lateral displacements of a railway bridge support is presented in [40]. The results of the simulation test of a cantilever beam indicate that the displacement could be accurately estimated even in the events where pseudo-static displacements due to non-symmetrical heavy train loading are dominant.

Based on the analysis of existing technologies, the main problems of the work have been defined. In the case of dynamic displacement monitoring, the main problem was considered to be the development of a method that would have an accuracy close to the existing methods using inclinometers designed for static applications and, at the same time, have no limitations, due to the limited possibilities of dynamic measurement using inclinometers designed for dynamic applications. The system has been developed mainly to determine the dynamic displacements associated with the passage of a train over a monitored bridge. Integrated signals from inclinometers and accelerometers recorded during the train’s passage are used to determine the dynamic movement. Signals from inclinometers are used to determine the so-called “quasi-static” component of displacement and the signal from the accelerometer to determine the dynamic component.

Signals from inclinometers before the train enters the bridge were used to determine static displacements. The course and results of the static test aimed to assess the temperature impact on the behavior of a given bridge structure at diurnal and long-term temperature changes, which particularly occurs in the case of steel arch bridges.

For this purpose, we use inclinometers designed for dynamic applications, which are, therefore, less accurate than those designed for static measurements. In the case of static displacement monitoring, the main problem was to investigate the possibility of using such inclinometers for static measurements and the need to verify these measurements with trigonometric surveys.

## 2. Description of Monitoring System

### 2.1. Hardware

The developed prototype of the monitoring system consists of a central unit and measurement modules [20]. Each module contains an 8-channel, 16-bit analog–digital converter adapted to support the following detectors:

- uniaxial gravity inclinometer with measuring range of ±1°;
- triaxial piezoelectric accelerometer with measuring range of ±5*g*;
- MEMS-type triaxial accelerometer with measuring range of ±3*g*;
- resistance strain gauge;
- temperature sensor with a measuring range from −55 °C to +125 °C.

The central unit, which controls the measuring module, consists of a microcontroller, local memory on an SD card, a real-time module, and a Global System for Mobile Communications (GSM) module. Communication with the measuring modules is established by means of the RS485 protocol. The system is equipped with an algorithm of detecting trains’ passages, thanks to which a loop of data acquisition is fully automated. The signals registered by the sensors during a train passage are sent as a text file to a remote file transfer protocol (FTP) server. The system works autonomously and the buffer power supply protects from power failures.

### 2.2. Algorithm and Software

**Figure 1:**
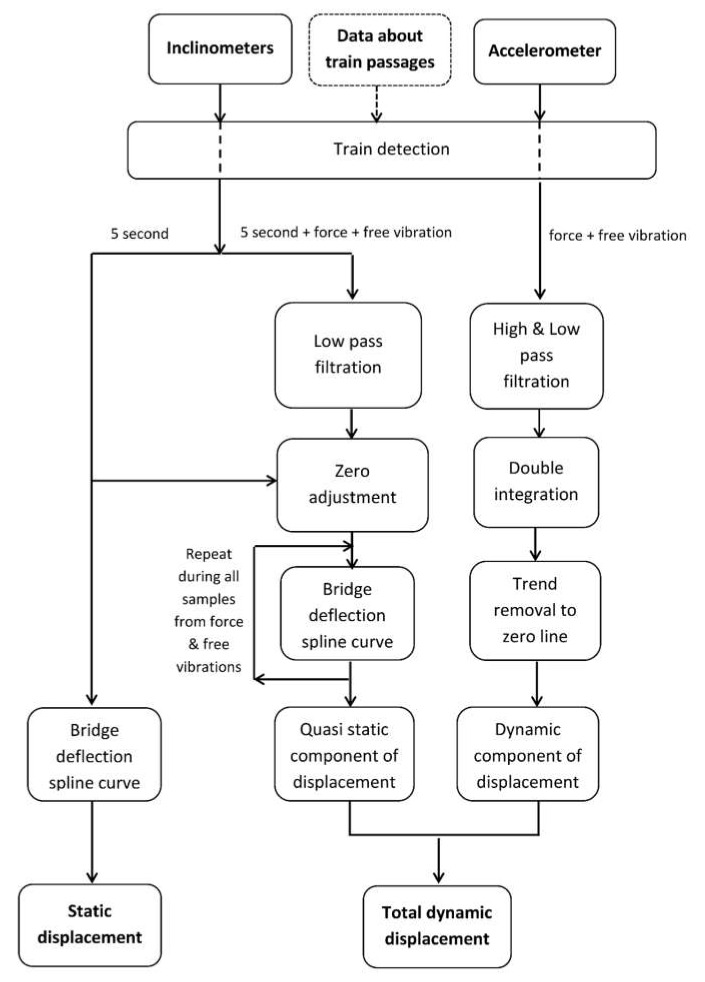
A flow chart of the static and dynamic displacement monitoring: The left path of the static displacement determination, the central path of the quasi static component, and the right path of the dynamic component of dynamic displacement determination.

Inclinometers are installed in one line on a bridge span and accelerometer at the point of displacement examination. Signals from the inclinometers are used to determine the static displacement before the train enters the bridge. During train passage, signals from the inclinometers are used to determine the so-called quasi-static component of a displacement, and the signal from the accelerometer is used to determine the dynamic component. A flow chart of the static and dynamic displacement monitoring is presented in Figure 1. In addition to data from inclinometers and the accelerometer, the system also uses external data about train passages: Type of multiple-unit trains or a separate locomotive and cars, gross weight, and length of the train. The data are supplied from the Department of Handling the Systems and Individual Timetables, Railway Traffic Management Centre of PKP PLK S.A. With respect to the safety requirements in the research and prototype solutions, the data are entered into the system in an off-line and semi-automatic mode. In the future, it will probably be possible to automatically download those data from the PKP PLK SA internal network while developing an application monitoring system.

The monitoring system measures all signals from the electronic sensors continuously, 24/7, with a 200 Hz sampling frequency. Due to restrictions on the size of data, the system records only the data corresponding to train passages. In the first stage of the monitoring of a single train passage, the approach of the train is detected and the recording of the measured signals is activated (5 s before entry) until the end of free vibrations (phase of the train detection at Figure 1).

Only after train descent, on the basis of analysis of the course from the inclinometer located as close as possible to train entry onto the bridge, the moment of the beginning and end of forced vibrations is determined. The signals registered by the electronic sensors include three parts:

(1) 5 s time registration of all signals before the train enters the bridge;
(2) force vibration registration when the train is on the bridge;
(3) free vibration registration after train leaves the bridge.

#### 2.2.1. Static Displacement Determination

The first part of the inclinometer signal registration (5 s) is used to calculate static displacements. The static displacements are determined in relation to the zero level when the inclinometers are installed. The method of determining the deflection line by means of spline curves is used [32,33,34,41]. This part of the signal processing is shown by the left path of the flow chart (Figure 1). Static displacements of the structure without train loading are mainly caused by temperature changes over some period (during a day and during a year) and can be compared to periodic trigonometric measurements.

**Figure 2:**
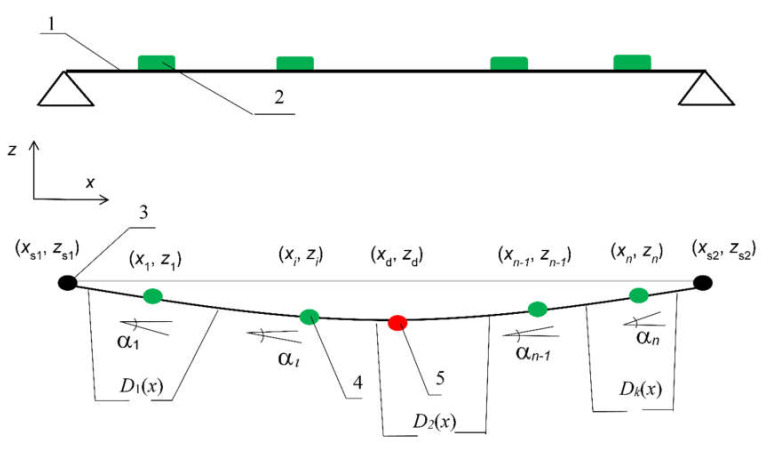
Diagram of the structure deflection line outlining: Upper—the arrangement of inclinometers (**1**) along the monitored span; (**2**) lower—the spline curve outlined, where: (**3**) Support point, (**4**) point of the inclinometer’s location, (**5**) point of the displacement examination; (*x*_s1_,*z*_s1_) and (*x*_i_,*z*_i_) respectively the coordinates of the span support points and inclinometers; *a_i_*, inclinometer readings; *D_k_*(*x*), spline curve.

The principle of determining the structure deflection lines using spline curves is shown in Figure 2.

In the unloaded state, we take a straight line as a reflection of the section under analysis along the structure with inclinometers placed. The displacement curves are determined in relation to the straight line. Input data are the coordinates *x_i_* of the inclinometer arrangement and the coordinates of the supports *(x*_s1_, *z*_s1_) and *(x*_s2_, *z*_s2_). For the sake of simplification, we assume zero coordinates of the first support: *x*_s1_ = 0, *z*_s1_ = 0, and the zero coordinate of the second support *z*_s2_ = 0. To determine the deflection line, we use indications of *α*_i_(t) with i of that inclinometer in the time function.

The vertical displacement curve comprises 3 order *D_j_*(*x*) spline curves, which represent the analyzed cross-section along the structure [34,42]:

(1) $$D_{j}\left(x\right)=\;d_{3,j}x^{3}+d_{2,j}x^{2}+d_{1,j}x+d_{0,i}$$

where *d*_3,*j*_, *d*_2,*j*_, *d*_1,*j*_, *d*_0,*j*_ are the expansion coefficients of the displacement curve function.

As nodes of spline curves *D_j_*(*x*), we assume support points, inclinometer installation points, and curve connection points. Due to unfavorable properties of spline curves to create an excessive number of inflection points, in the case of the measurement data of angles distorted by measurement errors, we try to minimize the number of spline curves.

In the presented example (Figure 2), three spline curves *D*_1_(*x*), *D*_2_(*x*), and *D_k_*(*x*) (*k* = 3) were used. The points of the location of inclinometers *i* = 2 and 3 were taken as the points of the curve connection.

For individual points, we create the following equations:

1. points of inclinometers’ location (coordinates *(x_i_*,*z_i_*)) with *D_j_*(*x*) curve:

(2) $$\;\;\tan\alpha_{i\_}=3d_{3,j}x_{i}{}^{2}+2d_{2,j}x_{i}+d_{1,j}\;,$$

2. points of connection of curves *D_j_*(*x*) and *D_j+_*_1_(*x*):

(3) $$3d_{3,i}x_{i}{}^{2}+2d_{2,i}x_{i}+d_{1,i}=3d_{3,i+1}x_{i}{}^{2}+2d_{2,i+1}x_{i}+d_{1,i+1}\;,$$

(4) $$6d_{3,i}x_{i}+2d_{2,i}=6d_{3,i+1}x_{i}+2d_{2,i+1}$$

(5) $$d_{3,i}x_{i}^{3}+d_{2,i}x_{i}^{2}+d_{1,i}x_{i}+d_{0,i}=\;d_{3,i+1}x_{i}^{3}+d_{2,i+1}x_{i}{}^{2}+d_{1,i+1}x_{i}+d_{0,i+1}$$

3. support points (coordinates *(x_s_*_1_, *z_s_*_1_) or *(x_s_*_2_, *z_s_*_2_)) belonging to curve *D*_1_(*x*) or *D_k_*(*x*):

(6) $$\begin{array}{l} z_{s}{}_{1}=d_{3}{}_{,1}x_{s}{{}_{1}}^{3}+d_{3}{}_{,1}x_{s}{{}_{1}}^{2}+d_{3}{}_{,1}x_{s}{}_{1}+d_{0}{}_{,1},\\ or\\ z_{s}{}_{2}=d_{3,k}x_{s}{{}_{2}}^{3}+d_{2,k}x_{s}{{}_{2}}^{2}+d_{1,k}x_{s}{}_{2}+d_{0,k}. \end{array}$$

For simple-supported structure support points, the following may be introduced:

(7) $$\begin{array}{l} \tan\alpha_{1}=3d_{3}{}_{,1}x_{s}{{}_{1}}^{2}+2d_{2}{}_{,1}x_{s}{}_{1}+d_{1}{}_{,1},\\ or\\ \tan\alpha_{n}=3d_{3}{}_{,k}x_{s}{{}_{2}}^{2}+2d_{2}{}_{,k}x_{s}{}_{2}+d_{1}{}_{,k}. \end{array}$$

The individual equations are based on the following conditions:

- tangent to the angle indicated by the inclinometer, according to Equation (2);
- smooth curves (first derivative), according to Equation (3);
- curves with continuous curvature (second derivation), according to Equation (4);
- curve continuity, according to Equation (5);
- a known (zero) coordinate of support *z_s_*, according to Equation (6);
- angle on the outer support equal to the indications of the nearest inclinometer, according to Equation (7).

For redundant data, the equation system created is solved via least-squares means. The final solution is then partly immune to possible errors associated with the inclinometer errors and the filtration method used. In the example shown in Figure 2, the static displacement *d*_s_ is calculated as a point of the *D*_2_(*x*_d_) spline curve:

(8) $$d_{s}=d_{3,2}x^{3}+d_{2,2}x^{2}+d_{1,2}x+d_{0,2}$$

In order to calibrate the inclinometer measurements of static vertical displacements with respect to the external coordinate system, an independent trigonometric survey is planned over long intervals. A group of prisms installed on the bridge span is observed using Total Station (TS) in serial mode with automatic target recognition. Each measurement consists of three full series with discrepancy control. Measurements are referenced to three fixed points—two close and one distant. The latter serves as a directional and refractive point. The aim of the survey is to determine the vertical deflections of the span. On the basis of a stable refraction point, an angle correction is calculated to minimize the effect of vertical refraction. Corrected and averaged vertical angles and distances are used to calculate displacements of the bridge in places where prisms are located.

#### 2.2.2. Dynamic Displacement Determination

All 3 parts of registered inclinometer signals (5 s time registration, forced and free vibrations) are used to calculate the quasi-static component *d_sc_*(*t*) of dynamic displacement *d_d_*(*t*). This part of the signal processing is shown at the central path of the flow chart (Figure 1).

In the first stage of the digital signal processing, the low-pass filtration is realized:

(9) $$\propto_{i}\left(t\right)\overset{low-pass\;filtration-F_{I\;CLP},\;n_{I\;L}\;\;}{\to }\propto_{i\;LP}\left(t\right)$$

where *α_i_*(*t*) is the inclinometer readings, *α_i LP_*(*t*) is the inclinometer readings after low-pass filtration, and *F_I CLP_* and *n_IL_* are the cut frequency and filter order, respectively.

Due to the determination of dynamic displacements in relation to the value of zero displacements before the train’s entry onto the bridge, based on the average of fragments of signals recorded $\Delta t_{5s}$ before the train’s entry, zeroing is carried out:

(10) $$\propto_{i\;ZLP}\left(t\right)=\propto_{i\;LP}\left(t\right)-\bar{\propto_{i\;LP}\left(\Delta t_{5s}\right)}$$

where *α_i ZLP_*(*t*)—inclinometer readings after low-pass filtration and adjustment to zero signal.

Next, the method of determining bridge deflection lines using spline curves (presented in 2.3.1) is applied, where values from inclinometer signals after filtration and zeroing are used as input angles $\propto_{i\;ZLP}\left(t\right)$.

The determination of successive spline curves *Dj*(*x*,*t*) is repeated to all samples from the inclinometer signals corresponding to forced and free vibrations. As in item 3.2.1, the quasi-static component of displacement versus time *d*_sc_(*t*) is calculated as

(11) $$d_{sc}\left(t\right)=c_{s}\times D_{2}\left(x_{d},\;t\right)$$

where *c*_s_ are scale coefficients of the quasi-static component.

To calculate the dynamic component *d_dc_*(*t*) of dynamic displacement *d_d_*(*t*), the accelerometer is located at the point of the displacement’s examination (Figure 2—point (*x*_d_,*z*_d_)). The forced and free vibration parts of the signal from the accelerometer are used for calculations (phase of the train detection at Figure 1). This part of the signal processing is shown in the right path of the flow chart (Figure 1).

In the first stage of the digital signal processing of the acceleration signal *a*(*t*), the high-pass and the low-pass filtrations are carried out:

(12) $$a_{i}\left(t\right)\overset{\;low\;\&\;high-pass\;filtration-\;\;F_{A\;CHP},\;n_{A\;H},\;\;F_{A\;CLP},\;n_{A\;L}\;}{\to }a_{i\;\;HLP}\left(t\right)$$

where *a_i_*(*t*) is the accelerometer readings, *a_i HLP_*(*t*) is the accelerometer readings after high- and low-pass filtration, and *F*_A CHP_, *F*_A CLP_, and *n*_AH_, *n*_AL_ are high- and low-pass cut frequencies, and filter orders, respectively.

In the second stage, the method of double integration is applied:

(13) $$d_{dI}\left(t\right)=\iint_{t_{1}}^{t_{2}}a_{HLP}\left(t\right)$$

where *d*_dI_(*t*)—result of double integration, *t*_1_—time of the beginning of the forced vibration, *t*_2_—time of the end of the free vibration.

Next, the method of removing the linear trend is applied:

(14) $$d_{dI}\left(t\right)\overset{\;removing\;linear\;trend\;\;}{\to }\;d_{dt}\left(t\right)$$

where *d_dt_*(*t*)—result of removing the linear trend.

At the last stage, the scale coefficients *c_d_* is applied:

(15) $$d_{dc}\left(t\right)=c_{d}\times d_{dt}\left(t\right)$$

The total dynamic displacement *d_d_*(*t*) caused by the train passage on the bridge is calculated as the sum of the quasi-static *d_sc_*(*t*) and the dynamic component *d_dc_*(*t*):

(16) $$d_{d}\left(t\right)=d_{sc}\left(t\right)+d_{dc}\left(t\right)$$

The method for the selection of filtration parameters for inclinometer and accelerometer signals and scale coefficients of final integration is presented in Section 3.

## 3. The First Tests

### 3.1. The Tested Bridge

**Figure 3:**
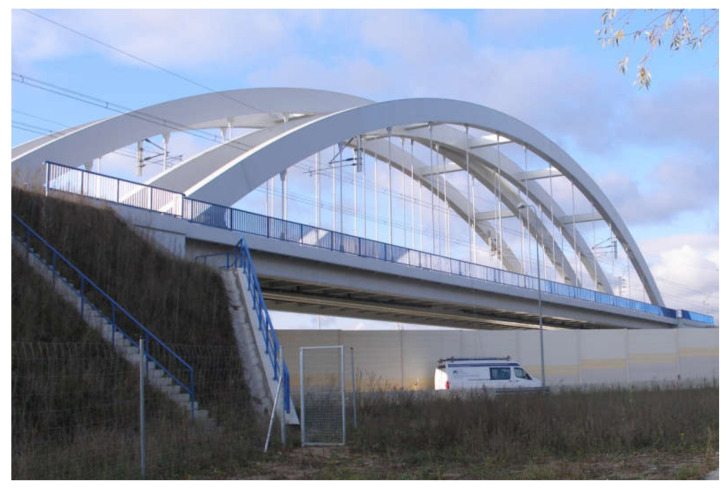
View of the tested bridge.

The system presented herein was implemented on an arch bridge located at a high-speed railway (Figure 3). The bridge consists of two new steel arch structures (from 2014 and 2015) with a span length of 75.00 m. The arch and the arch tie both have a box cross-section. The arch ties are suspended at the arches with the use of 13 pairs of steel hangers. The bridge has a ballasted deck, cross-bars from double-tee plate girders, and a reinforced concrete slab floor. The structure was accepted for train speeds up to 250 km/h.

In the case of the arch bridge, the extreme deflection appears at about one quarter of the span length (20 m from support). For preliminary testing, it was enough to monitor half of the span. The problem of optimal sensor olacement (OSP), particularly in applications for large structures, is not a trivial task and is combinatorial in nature. Optimization methods such as artificial neural networks (ANNs) [43], artificial bee colonies (ABCs) [44] or its extension—IABC [45], or genetic algorithms (GAs) [46] could be used. In the case of the tested bridge, a variation and extension of an iterative method, effective independence (EFI), was used. The details are presented in [47].

**Figure 4:**
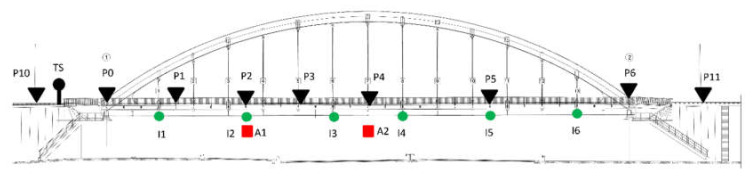
Diagram of inclinometer locations (I1, I2, and I3) and accelerometer location (A1) for permanent installation (one year monitoring), inclinometers (I4, I5, and I6) and accelerometer (A2) for short-term installation (one day test); prisms (the measuring: P0- P6, and the reference system: P10, P11, P12 are located outside the drawing); TS—Total Station location.

**Figure 5:**
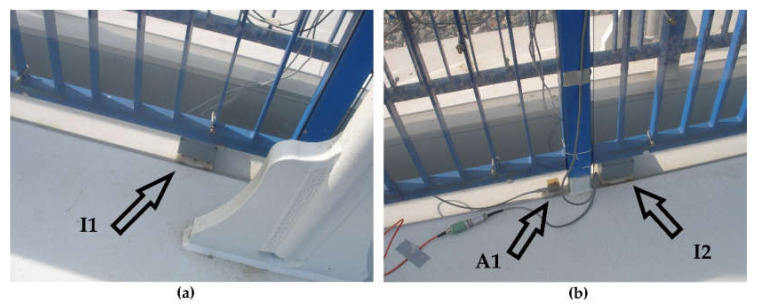
View of inclinometers and accelerometer location: (**a**) Inclinometer I1 location; (**b**) accelerometer A1, and inclinometer I2 location.

Three inclinometers were installed in one line at a bridge deck and the accelerometer was located at one quarter of a span length (Figure 4 and Figure 5). The installed monitoring system registers measurement data from all passing trains during one year.

Over the first 8 months of monitoring, on average once a month, the inductive transducer was installed at the extreme deflection point under various weather conditions to measure displacements caused by passages of various types of passenger trains: Separate locomotive and cars or multiple-unit trains. During comparative tests, 23 multiple-unit trains (19 ED250 and 4 ED160) and 19 separate locomotive and cars (18 EP09 and 1 EP07) were recorded.

Due to technical limitations, one day of additional comparison tests was carried out using an additional three inclinometers and one accelerometer (Figure 4). Three multiple-unit trains (2 ED250 and 1 ED160) and four separate locomotive and cars (EP09) were recorded.

**Figure 6:**
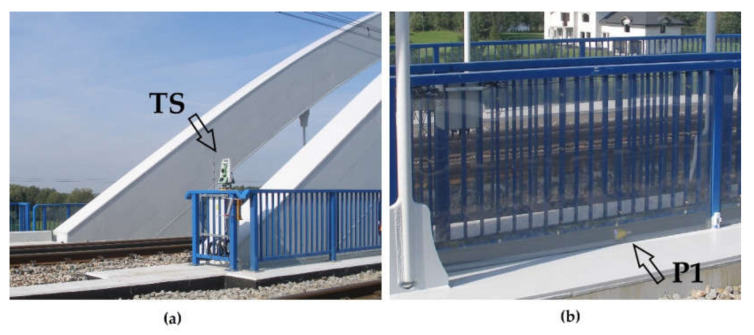
View of the trigonometric survey: (**a**) Total Station TS location; (**b**) prism P1 location.

A trigonometric survey was made using a robotic Total Station (TS) Leica TCRP1201+ (Figure 6a) and a set of prisms (Figure 6b). The method is based on determining the height differences of points signaled by prisms based on the quasi-continuous measurement of directions, vertical angles, and slope distances. The ordinates are calculated according to known relationships described, among others, in [48]. Observations of subsequent distances and angles are pre-corrected due to the influence of atmospheric factors in the manner described, among others, in [49], including the correction of apparent height changes due to atmospheric refraction. Due to the impossibility of forced installation of the instrument station in one place, it is necessary to calculate its current position using the free station method [48].

In the project in question, the instrument was mounted to the bridgehead at one side of the bridge (Figure 4 and Figure 6a) and was oriented parallel to the span. Two reference points were mounted on nearby traction poles and were used to control the position of the instrument. The refraction point was located on the opposite bridgehead. Each point was measured automatically in three series just before and after the train passage.

Due to small distances (up to 80 m), the impact of refraction was corrected with the linear equation: dβ = a·ΔT + b, where dβ is the correction of the vertical angle β, and ΔT—the difference between the air temperature during measurement and the reference temperature. For the needs of research, all-day observations were made to test the method of taking refraction into account and, independently, assessing changes in deflections of the span as a function of temperature.

For a temperature difference of 20 °C, the correction factors for the furthest points did not exceed 2 mm. Based on three series of observations, each time, an average error of the arithmetic mean was calculated for individual points—it ranged from ±0.1 to ±0.8 mm.

### 3.2. Algorithm Adaptation to the Tested Bridge

In the first stage, the accuracy of the structure quasi-static deflection mapping using spline curves was analyzed. The analyses were carried out using data from loading the numerical model of the structure with a fixed-formation train passing at the speed of 10 km/h. The obtained courses of angle changes were used to simulate the numerical determination of the bridge deflection curve using spline curves.

Analyses were carried out using four characteristic points (one support point and three inclinometer location points) and a different number of curves:

- a single 3° curve (designation 40),
- a single 3° curve with an angle of rotation at the support equal to that of inclinometer I1 (designation 41),
- two spline curves connected together at the point of location of inclinometer I2 with an angle of rotation at a support equal to that of inclinometer I1 (designation 42),
- three spline curves combined at the points of the locations of inclinometers I1 and I2 with an angle of rotation at a support equal to that of inclinometer I1 (designation 43).

**Figure 7:**
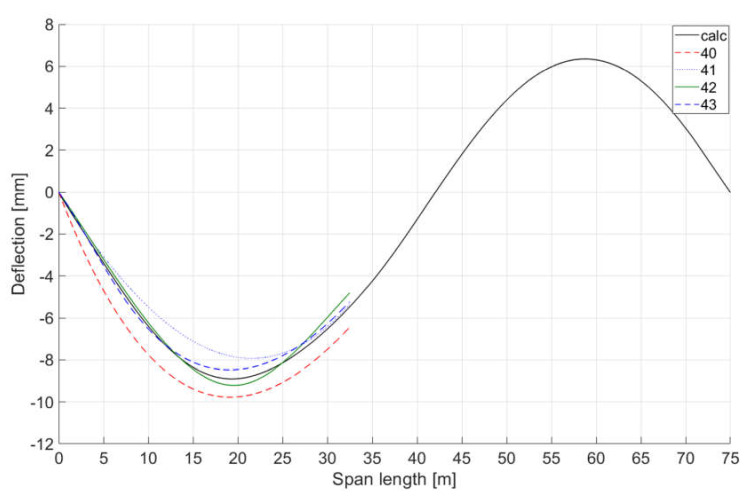
Analysis of the accuracy of the structure quasi-static deflection mapping using spline curves in relation to the deflection curve determined with the use of numerical model load data (calc); 40—deflection curve determined by a single 3° curve, 41—line determined by a single 3° curve with an angle of rotation at support equal to that of inclinometer I1, 42—line determined by 2 spline curves with an angle of rotation at support equal to that of inclinometer I1, 43—line determined by 3 spline curves with an angle of rotation at support equal to that of inclinometer I1.

**Figure 8:**
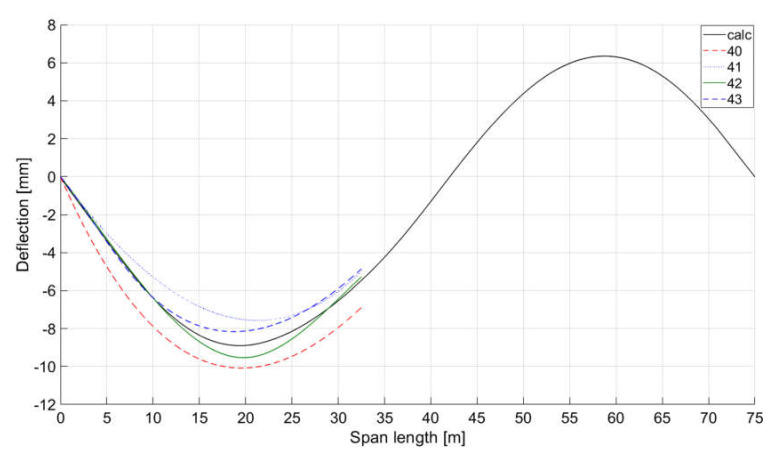
Analysis of the sensitivity to measurement errors of the structure quasi-static deflection mapping using spline curves in relation to the deflection curve determined with the use of numerical model load data (calc); 40—deflection curve determined by a single 3° curve, 41—line determined by a single 3° curve with an angle of rotation at support equal to that of inclinometer I1, 42—line determined by 2 spline curves with an angle of rotation at support equal to that of inclinometer I1, 43—line determined by 3 spline curves with an angle of rotation at support equal to that of inclinometer I1.

Figure 7 presents a comparison of the determined spline curves with the curve obtained from the numerical model. In the next analysis of the quality of spline curves determination, to the angles obtained from loading the numerical model of the train structure, disturbances simulating possible measurement errors in readings from inclinometers I1, I2, and I3 with a random distribution and maximum amplitude equal to 2.5% of the extreme numerically determined angle were added. The curves with the greatest deviations from the numerical curve are shown in Figure 8. The best results were obtained with three inclinometers at two spline curves. Similar simulations were performed using six inclinometers and four, five, and seven spline curves. The best results were obtained in this case with five spline curves.

In the second stage of the adaptation of the algorithm to the monitoring of a particular bridge structure, an analysis of the optimal selection of filtration parameters and scale coefficients of the final integration of quasi-static and dynamic components of the determined displacement was performed. Optimization was carried out with the criterion of the minimum average deviation (AD) of absolute relative deviations $\left|\Delta_{i\;min}/d_{r\;min}\right|\;$and $\left|\Delta_{i\;max}/d_{r\;max}\right|$, with two determined extreme deflection values in each run: Extreme downward *d*_d min_ (negative displacement and extreme value were marked “min”) and extreme upward (positive displacement and extreme value were marked “max”) from the respective deflection values measured with the reference method (inductive sensor) *d_r min_* and *d_r max_*:

(17) $$AD=\frac{\sum_{i=1}^{i=n\;}(\left|\frac{\Delta_{i\;min}}{d_{r\;min}}\right|+\left|\frac{\Delta_{i\;max}}{d_{r\;max}}\right|)\;}{2\times n}$$

(18) $$\Delta_{i\;min}=d_{d\;mini}-d_{r\;mini}$$

(19) $$\Delta_{i\;max}=d_{d\;maxi}-d_{r\;maxi}$$

where *i* is the train passage number and n is the number of passages analyzed.

**Figure 9:**
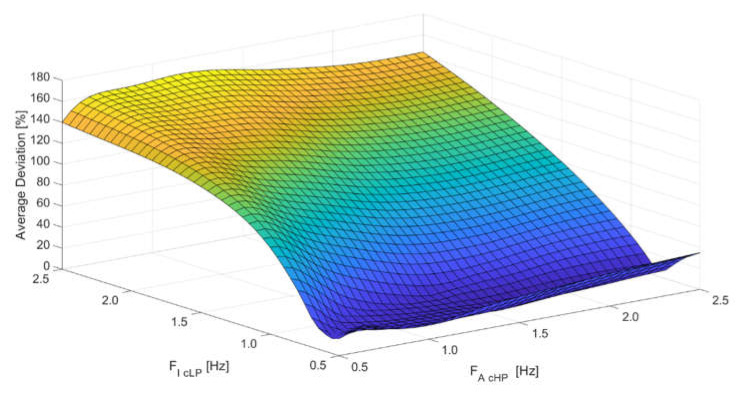
The distribution of average deviation at different cut-off frequencies of the *F*_I CLP_ low-pass filter signal from inclinometers and the *F*_A CHP_ high-pass filter signal from the accelerometer.

Optimization was carried out on the basis of six passages: three multiple-unit trains (ED250) and three separate locomotive and cars (EP09). The set of optimal filtration parameters and scale coefficients is shown in Table 1. An average deviation of 2.42% was obtained. Figure 9 shows the optimization of filtration parameters illustrated with the example of the distribution of average deviation values at different values of the low-pass filter *F*_I CLP_ cut-off frequency of signals from inclinometers and the high-pass filter *F*_A CHP_ cut-off frequency of signals from the accelerometer, with other parameters according to Table 1.

## 4. Results of the Bridge Monitoring

### 4.1. Static Displacement Measurement Results

The research aimed to harmonize the results of TS measurement with displacements calculated on the basis of measured inclinations including:

- tachymetric measurement before arrival of the train,
- 5 s inclinometer readings before the train reaches the bridge,
- calculation of displacement for both methods and comparison of results.

Readouts from the TS were initially analyzed for gross errors according to [50]. A difference from the average of more than three times the precision of the angle or distance measurement, i.e., ± 3 × 1″ or 3 × 1 mm, is used as the criterion for gross error. If this error is exceeded, the observation was rejected or measurement was repeated. Often, the total station misinterpreted readings to other prisms more than they were predefined. In addition, from time to time, it recorded observations significantly out of the others. All these cases were considered as outliers and were eliminated from further calculations. The remaining data were averaged and then adjusted due to the vertical displacements detected in the refraction point. Average errors of the measurement were also calculated.

The results from Total Station were compared to the static component from inclinometers measured before the train entry (5 s time registration of all signals before the train entry to the bridge). Analyses were conducted on the possibility of the use of dynamic inclinometers to measurements of static displacement.

**Figure 10:**
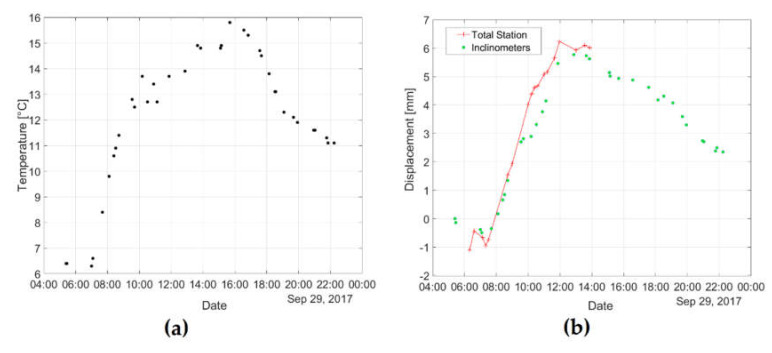
An example of an analysis of a one-day static displacement at ¼ span length point: (**a**) Average temperature measurements versus time; (**b**) indirect measurements of displacement with the use of inclinometer (green points) and total station measurements of displacement (red line with sharps) versus time.

**Figure 11:**
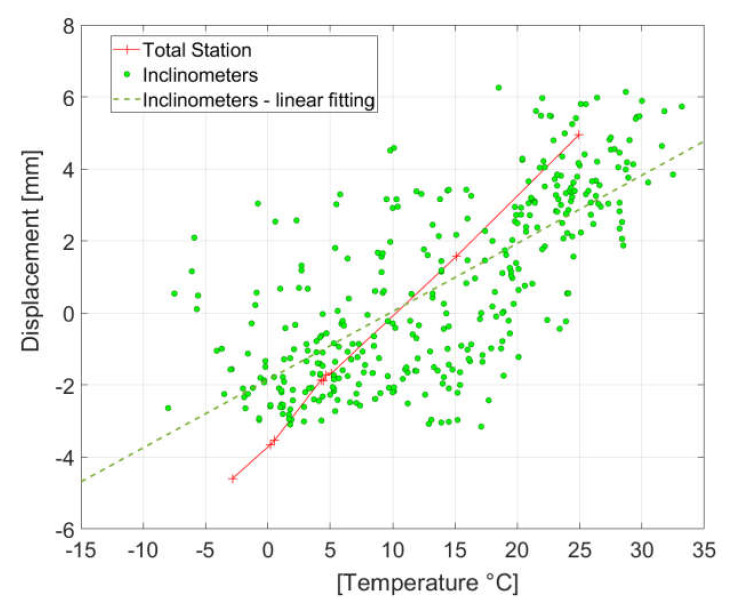
An example of an analysis of a one-year static displacement at ¼ span length point versus temperature; indirect measurements of displacement with the use of inclinometer (green points) with linear fitting (green dashed line) and total station measurements of displacement (red line with sharps).

The example of an analysis of static displacements during one day is presented in Figure 10 and during one year in Figure 11. The one-year registration contains the measurements done before all trains from 12 a.m. to 1 p.m. every day. Due to observed local deformations in the bridge deck as a result of temperature, it was not possible to determine static displacements based on the indications of three inclinometers in a manner analogous to determining the quasi-static curve described in point 2.3.1. The displacement at the ¼ span length point was determined based on the readouts of inclinometer No. 1 and 3 only.

In the case of one-day measurements, we can observe here a high compliance between an indirect survey of displacement with the use of inclinometer and TS measurements. Ten temperature measurement points were located uniformly on the bridge (at the steel structure and concrete plate) and the averaged value was assumed for the displacement comparison. In Figure 10, the consistency of changes in average temperature versus time (Figure 10a) with the displacement of the structure versus time (Figure 10b) is visible. However, influenced by insolation, the maximal temperature differences between measurement points at the same time were in the range of 3–8 °C. Due to these difficulties in temperature distribution assessment on the structure, it was not possible to obtain consistent numerical displacement results, and, therefore, the numerical results in Figure 10 and Figure 11 were not included.

In the case of one-year indirect measurements of displacement with the use of an inclinometer, we can observe a large dispersion of results versus temperature. After calculating the fitting line from the clouds of the inclinometer survey, the increase in displacement is equal to 2.0 mm for 10 °C, while 3.4 mm for 10 °C is determined on the basis of TS measurements. This is due to the high sensitivity of the structure deformations of the local and global nature to the changes in temperature. The resulting discrepancies will be the subject of further comparative analyses for both methods. In particular, research is conducted on a better refraction model for TS observations, taking into account the specificity of bridge measurements.

The observations during monitoring were confirmed by the numerical analyses of the structure deformations resulting from the changes in temperature. Numerical simulations [51] have shown, depending on temperature operating zone (modeled sun direction), that different modes have been obtained:

- uniform change in the temperature of the bridge (steel structure and concrete plate) generates mainly longitudinal deformation;
- temperature change of steel structure (excluding concrete plate) causes bending mode with 6.4 mm for 10 °C at the middle of the span length;
- temperature change of lateral wall of steel girder creates torsional deformation of the bridge.

### 4.2. Dynamic Displacement Measurement Results—Single Train Passages

**Figure 12:**
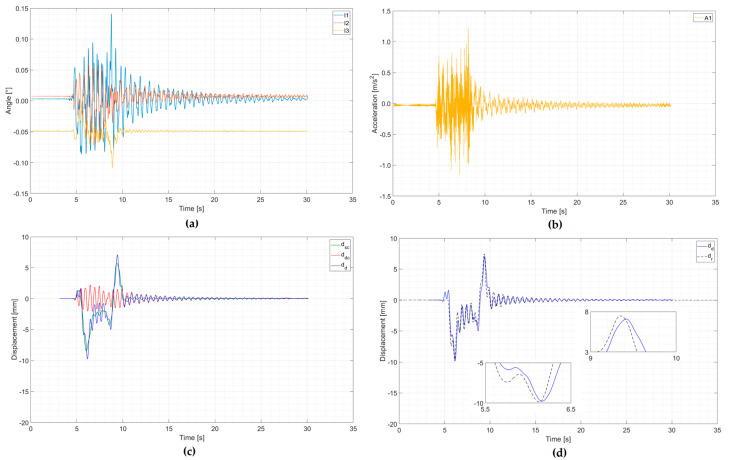
An example of an analysis of a multiple-unit train passage with a speed of about 190 km/h: (**a**) Signals from inclinometers; (**b**) signal from an accelerometer; (**c**) determined quasi-static displacement component (from inclinometers) *d*_sc_, dynamic displacement component (from an accelerometer) *d*_dc_, and total dynamic displacement *d*_d_; (**d**) comparison of total dynamic displacement *d*_d_ with reference measurement *d*_r_ (zoom of max and min values).

**Figure 13:**
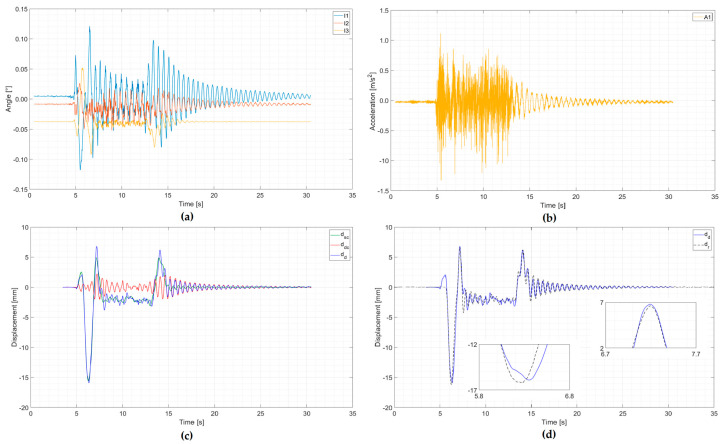
An example of an analysis of a separate locomotive and passenger cars passage with a speed of about 138 km/h: (**a**) Signals from inclinometers; (**b**) signal from an accelerometer; (**c**) determined quasi-static displacement component (from inclinometers) *d*_sc_, dynamic displacement component (from an accelerometer) *d*_dc,_ and total dynamic displacement *d*_d_; (**d**) comparison of total dynamic displacement *d*_d_ with reference measurement *d*_r_ (zoom of max and min values).

The example of an analysis of signal processing from a multiple-unit train is presented in Figure 12 and from a separate locomotive and cars in Figure 13. The first registration concerned a passenger multiple-unit train passage with a speed of about 190 km/h, consisting of an ED250 train, gross weight of 445 tons, and length of 187 m. The second registration concerned a separate locomotive and passenger cars passage with a speed of about 138 km/h, consisting of an EP09 locomotive, gross weight of 536 tons, and length of 294 m. In both examples, train data (type of the train, weight, and length) were obtained from the Railway Traffic Management Centre.

In both figures, the first two graphs present signals registered by the inclinometers (a) and accelerometer (b). The next graph (c) presents the method of integrating the signals from the inclinometers (a quasi-static component of the displacement) and from the accelerometer (a dynamic component) and total displacements calculated as a sum of the quasi-static and the dynamic component. The last graph (d) presents the comparison of total dynamic displacement with reference measurement.

The courses shown in Figure 12 and Figure 13 are characterized by a low level of extreme deviation from the reference measurements:

- Multiple-unit train: ∆*_min_* = 0.13 mm, ∆*_min_*/*d_r min_*= 1.3%, ∆*_max_* = 0.36 mm, ∆*_max_*/*d_r max_*= 4.8%;
- Separate locomotive and cars: ∆*_min_* = 0.36 mm, ∆*_min_*/*d_r min_*= 2.2%, ∆*_max_* = 0.22 mm, ∆*_max_*/*d_r max_*= 3.3%.

It should be noted that in both cases, there is also a high compatibility of local extremes of forced and free vibrations. These two passages did not belong to the group of six passages selected to optimize signal processing parameters from inclinometers and the accelerometer.

On the basis of all the passages, root-mean-square deviation (error)—the equivalent of the standard deviation *s_max_*, *s_min_* to the extreme minimum and maximum values—was determined from comparative studies:

(20) $$s_{min}=\sqrt{\frac{\sum_{i=1}^{i=n}{\left(d_{d\;mini}-d_{r\;mini}\right)}^{2}}{n-1}}$$

(21) $$s_{max}=\sqrt{\frac{\sum_{i=1}^{i=n}{\left(d_{d\;maxi}-d_{r\;maxi}\right)}^{2}}{n-1}}$$

where *n*—number of passages.

The results of the analysis of all passages and a separate locomotive and cars or multiple-unit trains are shown in Table 2.

**Figure 14:**
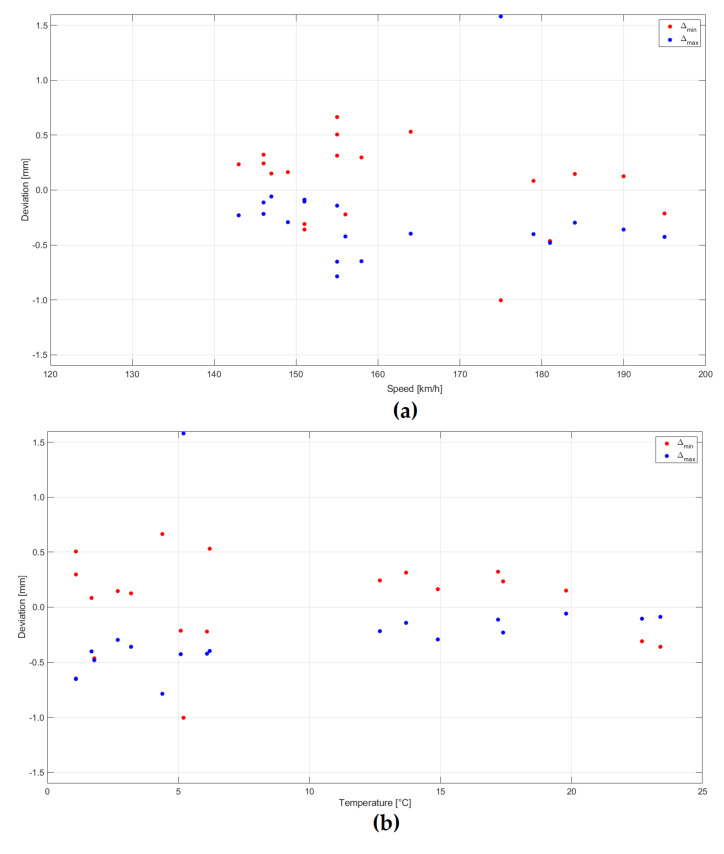
Analysis of the distribution of the measurement result deviation of extreme values in relation to reference measurements for multiple-unit trains (ED250) passages: (**a**) Depending on train speed and (**b**) air temperature.

**Figure 15:**
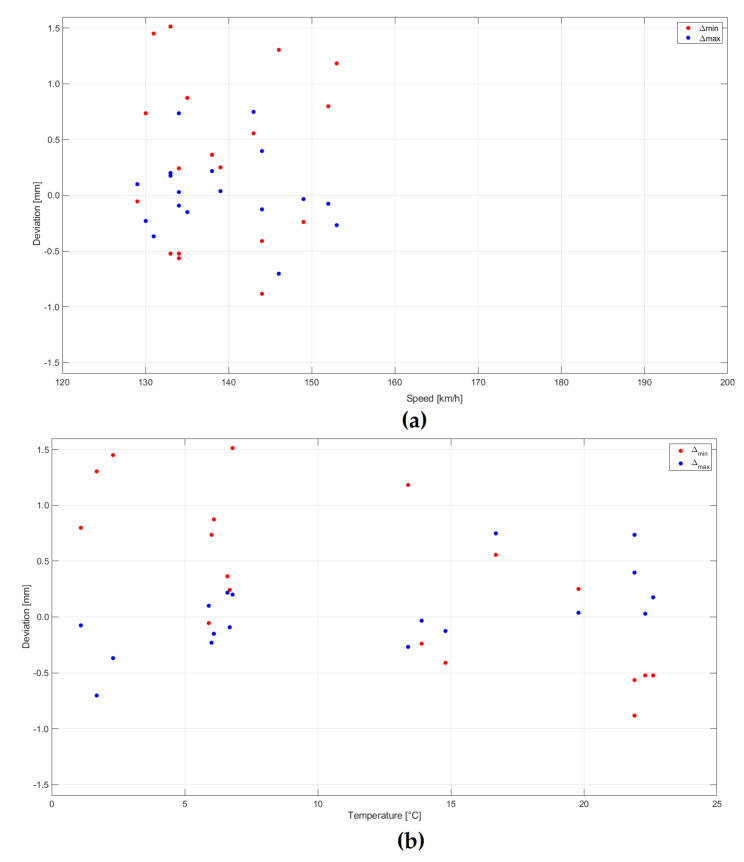
Analysis of the distribution of the measurement result deviation of the extreme values from the reference measurements for separate locomotive (EP09) and cars passages: (**a**) Depending on train speed and (**b**) air temperature.

Analyses of the distribution of deviation of measurement results of extreme values in relation to reference measurements were carried out, depending on the train speed and weather conditions—air temperature. Results are presented for multiple-unit trains in Figure 14 and for separate locomotive and cars passages in Figure 15. The bigger scatter of deviation values occurs in the case of separate locomotive and cars passages than multiple-unit trains. In both cases, there is no clear dependence of the magnitude of deviations on train speed. In the case of multiple-unit trains, most of the passages were made at speeds in the range of 150–160 km/h, and this is most probably the reason for bigger deviations at such speeds. From the graphs, it can be concluded that larger deviations occurred at lower air temperatures 0–5 °C.

Before possible subsequent tests, it would be worthwhile to carry out laboratory tests of the accuracy of inclinometer and accelerometer indications at different air temperatures.

On the basis of the tests carried out with the use of six inclinometers and a limited range of registered passages, it can be stated that by using five spline curves to determine quasi-static displacements in the case of displacements at the point of installation of the A1 accelerometer (Figure 4), the measurement results have changed significantly.

Deviation changes (from 40% to 1440%) were observed in relation to the deviations determined in relation to reference measurements with the use of three inclinometers. These deviations were within the acceptable range and did not exceed the standard deviation values from Table 2 (they were within the range of 1–86% *s_min_* or *s_max_*).

In the case of displacements at the A2 accelerometer installation point, relative deviations in relation to reference measurements assumed values within the range of 6–39%. It should be noted that extreme negative displacements were equal to about 50% of displacements from point A1. Extreme positive displacements were not considered, because in A2, they were close to zero. After applying different signal processing parameters than those given in Table 1, much smaller deviations were achieved. This indicates the necessity to select different signal processing parameters when determining the displacements at different points of the structure.

### 4.3. Dynamic Displacement Measurement Results—Continuous Monitoring

**Figure 16:**
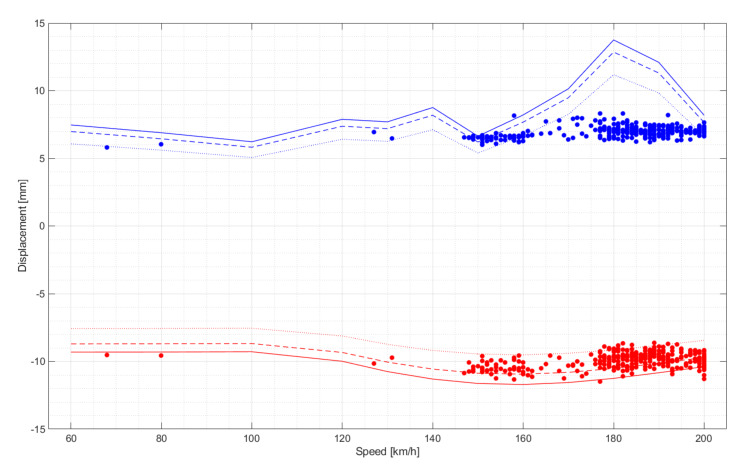
An example of displacement monitoring under the load of multiple-unit trains ED250 for one month (December 2017): Extreme displacement versus speed; red dot—minimum, blue dot—maximum; three red and blue lines correspond to the numerically determined extreme deflections from an empty train (dotted line), the state of normal use (dashed line), and the overloaded state foreseen by the manufacturer (solid line).

The installed monitoring system registered data on all trains passing from 1st of June 2017 to 31th of May 2018. The displacement values determined by the monitoring system were compared to the values determined using the numerical modeling [47]. Numerical analyses were performed especially for multiple-unit trains (ED250—popular name Pendolino). It was the only type of train allowed to run at speeds up to 200 km/h or more during the project. The remaining passenger trains were running at a maximum speed of 160 km/h. Figure 16 shows the relationship between the extreme negative and positive displacements recorded by the monitoring system during the multiple-unit trains ED250 in December. This was the first month in which the permitted speed was raised from 160 to 200 km/h. It should be noted that due to time reserves in the timetable and the proximity of the destination station, not all trains were running at maximum speed. The figure shows three lines each corresponding to the numerically determined extreme deflections from an empty train, the normal state of use, and the overloaded state foreseen by the manufacturer.

In the case of extreme minimum values, there is a high compliance of the measured and numerically determined values in the speed range from 60 to 200 km/h. The maximum differences slightly exceed twice the standard deviation. For extreme maximum values in the speed range above 150 km/h, there is a distortion in the calculated displacements based on the numerical model. The structure load of a moving train was modelled by means of a moving system of concentrated forces corresponding to the static axial load of the train. This approach is a simplification of the modelled inertial forces and may distort solutions for higher travel speeds. Detailed analyses and comparisons of the results of monitoring and numerical analyses will be published separately. The developed numerical 3D model was prepared based on a technical documentation using Abaqus software and calibrated for testing train passages with different speeds. Detailed information about this model applied to the sensor placement problem, as well as comparison responses (computed and measured) for selected train passages, can be found in [51]. A versatility of numerical simulations of structural vibrations has not only facilitated the algorithm development for vertical displacement assessment but also makes the possibility for verification of the proposed approach.

## 5. Conclusions

This article presents a bridge structure monitoring system whose main elements are inertial transducers used to determine vertical displacements using an indirect method. The elaboration of the data from inclinometers together with an accelerometer for indirect displacement measurement under the dynamic load is the main achievement of the system.

Studies have confirmed the need for periodic measurements of total station for calibration and verification of static readings from inclinometers. This should also be considered using other measurement methods, for example, time-synchronized, periodic photogrammetric measurements could also be useful to verify static displacements determined from dynamic measurements.

Tests of the system carried out so far proved its usefulness for monitoring bridges in a high-speed railway (up to 200 km/h), as well as their possibility to achieve high accuracy while determining dynamic displacements using an indirect method. It should be noted that this accuracy is close to or better than those of other measurement methods designed for continuous monitoring and not requiring reference points. It has been confirmed in long-term tests on a railway bridge in operation. This was the primary purpose of the presented tests.

The proposed system of optimization of the signal processing parameters requires comparative measurements at the beginning. These measurements must be carried out at all deflection monitoring points. The displacement courses obtained after its application are characterized by the relatively high accuracy of the extreme values determined, such as reconstruction of the shape of the forced vibration course during train passage like free vibration after train exit from a bridge.

It seems to be advisable to carry out further works connected with finding the reasons for much bigger measurement deviations in the case of some train passages. It seems advisable to develop an algorithm with the auto-modification of signal processing parameters on the basis of analysis of recorded signals from inclinometers and the accelerometer, e.g., based on their spectral analysis. Developing a method of algorithm parameters auto-modification could lead to a reduction in the number of measurements with much larger deviations than the others. Thanks to such an auto-modification of the algorithm, it might also have been unnecessary to make comparative measurements at many points of the structure, in the phase of launching a monitoring system at a new structure.

The presented monitoring system was installed and tested on the single-line railway bridge; thus, the loading generated by passing trains induced mainly the bending mode of the structure. An application of the monitoring system to multi-line railway structures seems to also be possible; however, additional transducers may be required to identify and quantify the influence of twisting modes for vertical displacement assessment of the structure.

## Figures and Tables

**Table 1:** The set of optimal parameters of the signals processing.

Type of the Signal Processing	Inclinometers	Accelerometer
High-pass filtration	*---*	*F*_A CHP_ = 1.67 Hz *n*_AH_ = 6
Low-pass filtration	*F*_I CLP_ = 0.73 Hz *n*_IL_ = 4	*F*_A CLP_ = 30 Hz *n*_AL_ = 6
Integration	*c*_s_ = 1.01	*c*_d_ = 1.30

**Table 2:** Root-mean-square deviation for all trains, multiple-unit trains, and separate locomotive and cars passages.

Train	Root-Mean-Square Deviation			
	*s_min_*	*s_max_*	*s_min_/d_r min_*	*s_max_/d_r max_*
	[mm]	[mm]	[%]	[%]
All trains	0.64	0.53	4.8%	8.2%
Multiple-unit trains ED250	0.41	0.54	3.8%	7.5%
Separate locomotive EP09 and cars	0.84	0.36	4.9%	6.0%
